# A transformer based deep learning framework for accurate single nucleotide variant correction in heterogeneous samples

**DOI:** 10.3389/fmicb.2026.1838029

**Published:** 2026-05-20

**Authors:** Xiaonan Wang, Shenjie Wang, Zhili Chang, Minchao Zhao, Xian Zhang, Nazarov Fayzullo, Eshtemirov Bunyod, Shuotong Li, Jiayin Wang

**Affiliations:** 1 School of Computer Science and Technology, Xi'an Jiaotong University, Xi'an, China; 2 Shaanxi Engineering Research Center of Medical and Health Big Data, Xi'an Jiaotong University, Xi'an, China; 3 Nanjing Geneseeq Technology Inc., Nanjing, Jiangsu, China; 4 Department of Respiratory Medicine, The Second Affiliated Hospital of Xi'an Jiaotong University, Xi'an, China; 5 AI and Digital Technologies Faculty, Samarkand State University, Samarkand, Uzbekistan; 6 College of Economics, Shenzhen University, Shenzhen, China

**Keywords:** genomic profiling, heterogeneous samples, host-microbe symbiosis, single nucleotide variant correction, transformer architecture

## Abstract

Profiling host genetic variations in heterogeneous host-microbiome mixtures is crucial for understanding cross-species interactions and microenvironmental dynamics. However, the variable host DNA fraction (purity) in bulk sequencing data severely compromises the performance of standard variant callers, leading to significant systematic biases in quantifying single nucleotide variants (SNVs). To address this, we developed a Transformer-based computational framework designed to model sequence context and technical artifacts in low-purity samples. The architecture employs a group-encoding mechanism to process multidimensional features—including variant allele frequency (VAF) distributions, base-level purity estimates, sequencing depth, and local genomic context (such as repeat regions and chromatin accessibility). By capturing long-range dependencies among these diverse signals, the model effectively neutralizes purity-induced biases to accurately recover the true host SNV count. We evaluated the framework using simulated sequencing data across a broad purity gradient (0.2–1.0). Our approach significantly reduced quantification errors, achieving high concordance between the corrected and actual ground-truth SNV counts. Benchmarking the corrected counts against the raw outputs of conventional callers (Mutect, Freebayes, LoFreq, and Platypus) demonstrated substantial performance gains, particularly in ultra-low purity conditions (0.2–0.3) where traditional statistical priors typically fail to provide reliable quantifications. Feature ablation and residual analyses further validated the independence of the multidimensional inputs and the unbiased, zero-centered nature of the count corrections. This deep learning pipeline provides a robust solution for the accurate quantification of host SNVs in complex biological mixtures, facilitating reliable downstream genetic analyses in highly heterogeneous microenvironments.

## 1 Introduction

Microbial communities form intricate symbioses with their hosts across diverse ecological niches, driving ecosystem resilience and functional homeostasis (Sommer and Bäckhed, 2013; Bulgarelli et al., 2013; McFall-Ngai et al., 2013). In host-associated environments, such as the gut mucosa or plant root systems, these dynamic interactions are fundamentally shaped by the genetic landscapes of both the host and the resident microbiota (Blekhman et al., 2015; Goodrich et al., 2014). To dissect the molecular mechanisms underlying these partnerships, researchers increasingly utilize high-throughput bulk sequencing to profile the entire microenvironment (Goodwin et al., 2016; Quince et al., 2017). However, these tissue samples are inherently heterogeneous, comprising a complex admixture of host cells and diverse microbial taxa (Nayfach and Pollard, 2016).

A primary computational challenge in analyzing these mixed-species sequencing data is the accurate quantification of host genetic variations, particularly single nucleotide variants (SNVs; Nielsen et al., 2014; Sunagawa et al., 2013). In such heterogeneous samples, the host DNA fraction fluctuates drastically depending on the microbial load and sampling site. Conventional variant callers [such as Mutect (Cibulskis et al., 2013), Freebayes (Garrison and Marth, 2012), Platypus (Rimmer et al., 2014), and LoFreq (Wilm et al., 2012)] rely on statistical priors optimized for relatively pure or clonal samples. When applied to host-microbiome mixtures with moderate to extremely low host purity, these algorithms suffer significant performance degradation (Alioto et al., 2015; Hwang et al., 2015), leading to substantial systematic biases in SNV quantification. This analytical limitation restricts the reliable assessment of host genetic factors and their subsequent impact on the symbiotic ecosystem (Benson et al., 2010).

To address the limitations of existing variant calling pipelines in low-purity contexts, we developed a Transformer-based deep learning framework to accurately quantify SNVs in heterogeneous samples (Poplin et al., 2018). Built upon the Transformer architecture (Ashish et al., 2017), our model incorporates a multi-dimensional feature extraction module, a group encoder, and a decoding layer. By systematically integrating diverse genomic and sequencing features—including variant allele frequency (VAF), sequencing depth, repeat region mapping, DNase hypersensitivity profiles, and genomic blacklist annotations (Amemiya et al., 2019)—the model captures the complex, non-linear error distributions driven by low purity and mapping artifacts.

In this study, we evaluated the proposed framework using simulated sequencing datasets that model the varying purity levels characteristic of host-microbiome mixtures. Our findings demonstrate that the Transformer-based pipeline effectively neutralizes purity-induced biases, accurately recovering the true host SNV counts. Comparative benchmarking against standard variant callers demonstrates that our tool yields superior quantification accuracy across all purity gradients (0.2–1.0), maintaining exceptional robustness even in ultra-low purity conditions. By providing a statistically sound computational solution for host SNV quantification in complex biological mixtures, this framework enables more precise investigations into interspecies genetic interactions.

## 2 Materials and methods

### 2.1 Overall framework of the SNV correction model

**Figure 1:**
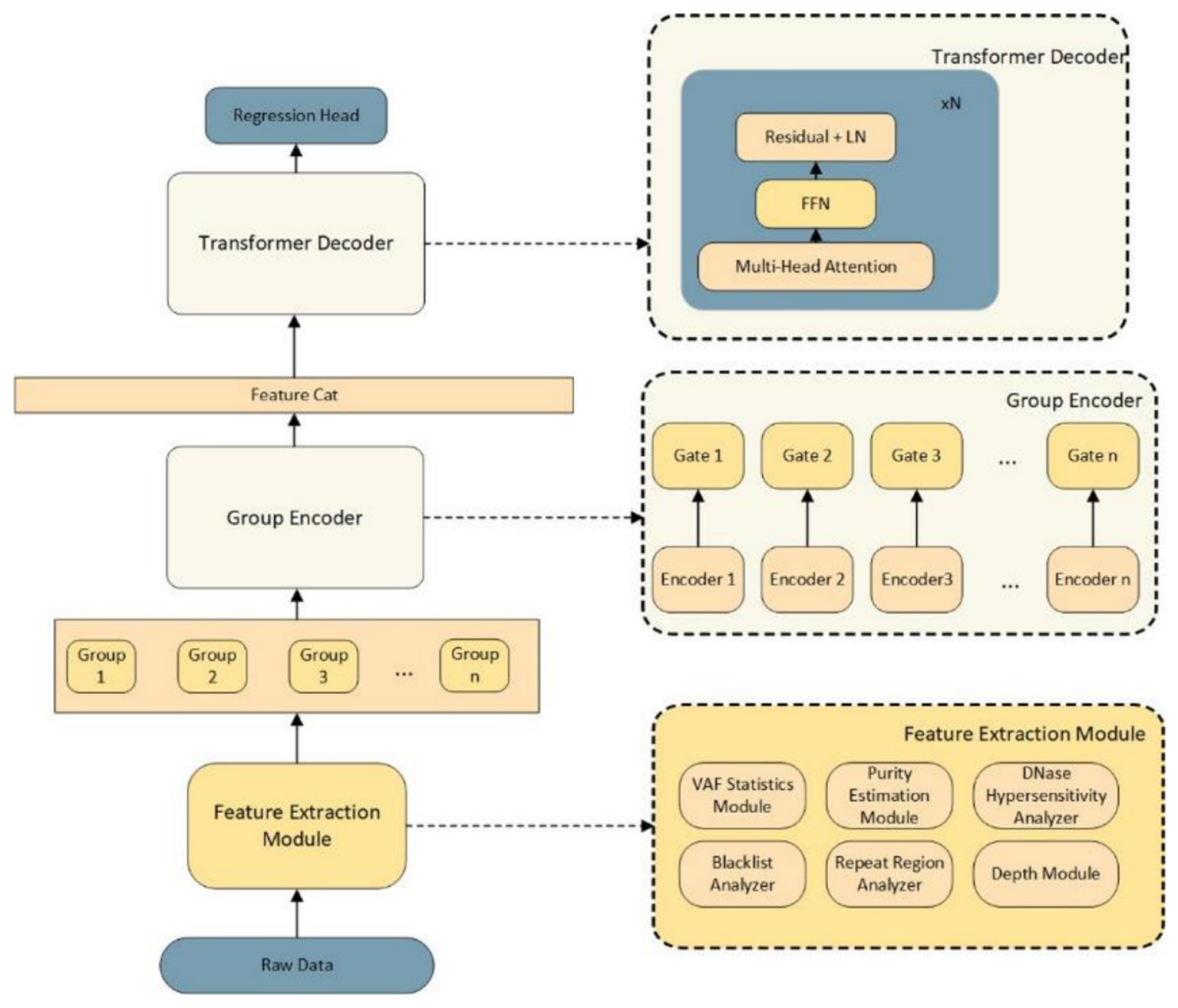
The overall architecture of the transformer-based SNV correction framework. The model is designed to process heterogeneous sequencing data from host-microbiome mixed samples. It consists of three main components: **(bottom)** a Feature Extraction Module that systematically captures multi-dimensional characteristics including VAF, purity, and genomic context; **(middle)** a Group Encoder with independent sub-encoders and gating mechanisms for dynamic feature integration; and **(top)** a Transformer Decoder that models complex long-range dependencies to output the finely calibrated SNV count via a Regression Head.

To address the inaccuracy of SNV detection in heterogeneous host-microbiome samples, we developed a deep learning framework based on a Transformer architecture. The model is designed to learn the complex error distributions associated with varying host DNA purities and sequencing artifacts, thereby outputting a corrected, highly accurate SNV count. The overall architecture of the proposed method is illustrated in Figure 1.

The framework processes raw sequencing data through a bottom-up pipeline, consisting of three core computational components: a Feature Extraction Module, a Group Encoder, and a Transformer Decoder, ultimately culminating in a Regression Head for the final SNV count prediction.

### 2.2 Feature extraction and grouping strategy

The accuracy of variant calling in mixed samples is heavily influenced by genomic context and sequencing quality. As shown in the lower section of Figure 1, our Feature Extraction Module systematically extracts multi-dimensional characteristics from the raw data. To ensure the model captures distinct biological and technical signals without overwhelming the attention mechanisms, we categorized these features into specific functional modules:

(1) VAF Statistics Module: Captures the Variant Allele Frequency distribution, which is highly sensitive to the host's sample purity.
(2) Purity Estimation Module: Provides a baseline estimation of the host DNA fraction in the symbiotic mixed sample.
(3) Genomic Context Analyzers: Includes the DNase Hypersensitivity Analyzer, Repeat Region Analyzer, and Blacklist Analyzer to account for regions prone to sequencing errors, mapping artifacts, or structural complexities.
(4) Depth Module: Evaluates the sequencing depth, a critical factor for distinguishing true variants from background noise in low-purity settings.

These extracted features are organized into distinct groups (Group 1, Group 2, …, Group *n*) to maintain their contextual integrity before passing them to the encoding layer.

### 2.3 Group encoding and feature concatenation

To process the grouped features effectively, we designed a Group Encoder (middle section of Figure 1). Each feature group is processed by an independent sub-encoder (Encoder 1 to Encoder *n*) followed by a gating mechanism (Gate 1 to Gate *n*). This architecture allows the model to independently weigh the importance of different feature sets—for instance, dynamically increasing the weight of the VAF and Purity features when dealing with highly heterogeneous (low-purity) samples. The outputs from these gates are then integrated through a Feature Concatenation (Feature Cat) layer, creating a unified, high-dimensional representation of the sample's sequencing profile.

### 2.4 Transformer decoder and regression output

The concatenated features are fed into the Transformer Decoder (upper section of Figure 1), which serves as the core reasoning engine of our framework. Comprising N stacked blocks—each containing Multi-Head Attention, Feed-Forward Networks (FFN), and Residual connections with Layer Normalization (Residual + LN)—the decoder excels at identifying long-range dependencies and complex correlations among the diverse feature groups (e.g., how sequencing depth interacts with repeat regions under specific purity conditions). Finally, the context-rich embeddings generated by the Transformer Decoder are passed to the Regression Head, which outputs the continuous numerical value representing the corrected actual SNV count for the host genome.

### 2.5 Simulated dataset generation for symbiosis research

To train and evaluate our framework, we generated synthetic sequencing datasets that mimic the complex microenvironments of host-microbiome symbioses. We computationally mixed host genomic data sourced from the database with varying proportions of sequences to create a gradient of host sample purities (ranging from 0.2 to 1.0). This simulation strategy strictly controlled the actual SNV counts, enabling a precise calculation of detection residuals and providing a robust benchmark against traditional variant callers.

## 3 Results

### 3.1 Overall efficacy of SNV count correction in heterogeneous samples

The primary objective of our Transformer-based framework is to rectify the systematic biases in SNV detection caused by the high heterogeneity of host-microbiome mixed samples. To evaluate the overall correction efficacy, we compared the raw SNV counts detected by standard pipelines, the corrected counts generated by our model, and the actual true counts (ground truth) across various simulated sample IDs.

**Figure 2:**
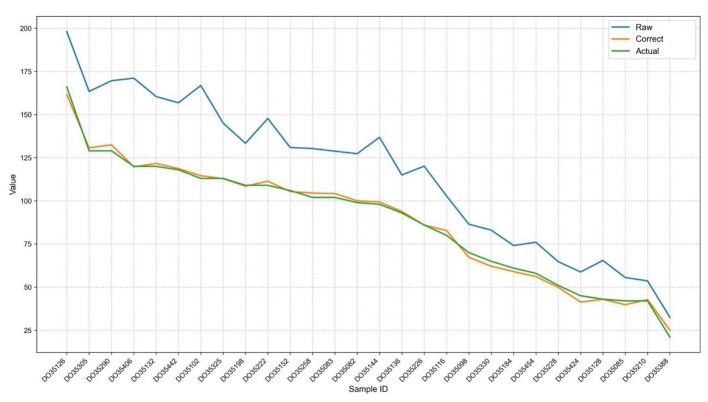
Overall efficacy of the SNV correction model. The line chart illustrates the comparison between the raw SNV counts (blue line) detected by standard pipelines, the actual true counts/ground truth (green line), and the model-corrected counts (orange line) across various simulated sample IDs. The substantial alignment between the corrected and actual trajectories demonstrates the model's ability to neutralize purity-induced biases.

As illustrated in Figure 2, the raw SNV counts (blue line) exhibited significant deviation from the actual true counts (green line), generally overestimating or underestimating the variants due to sequencing noise and varying host DNA fractions. However, after processing through our deep learning framework, the corrected SNV trajectory (orange line) demonstrated a remarkable alignment with the actual counts. The gap between the raw and actual values was substantially minimized, indicating that our model successfully learned the underlying noise patterns and effectively calibrated the SNV quantification for heterogeneous samples.

### 3.2 Benchmarking against state-of-the-art callers across purity gradients

In symbiotic microenvironments, the proportion of host cells can vary dramatically. Therefore, a robust computational tool must maintain high accuracy across a wide range of sample purities. We benchmarked our framework against four widely used variant callers: Mutect, Freebayes, Platypus, and LoFreq. The performance was evaluated across five distinct host purity ranges: 0.8–1.0, 0.7–0.8, 0.5–0.7, 0.3–0.5, and the highly challenging ultra-low purity range of 0.2–0.3.

**Figure 3:**
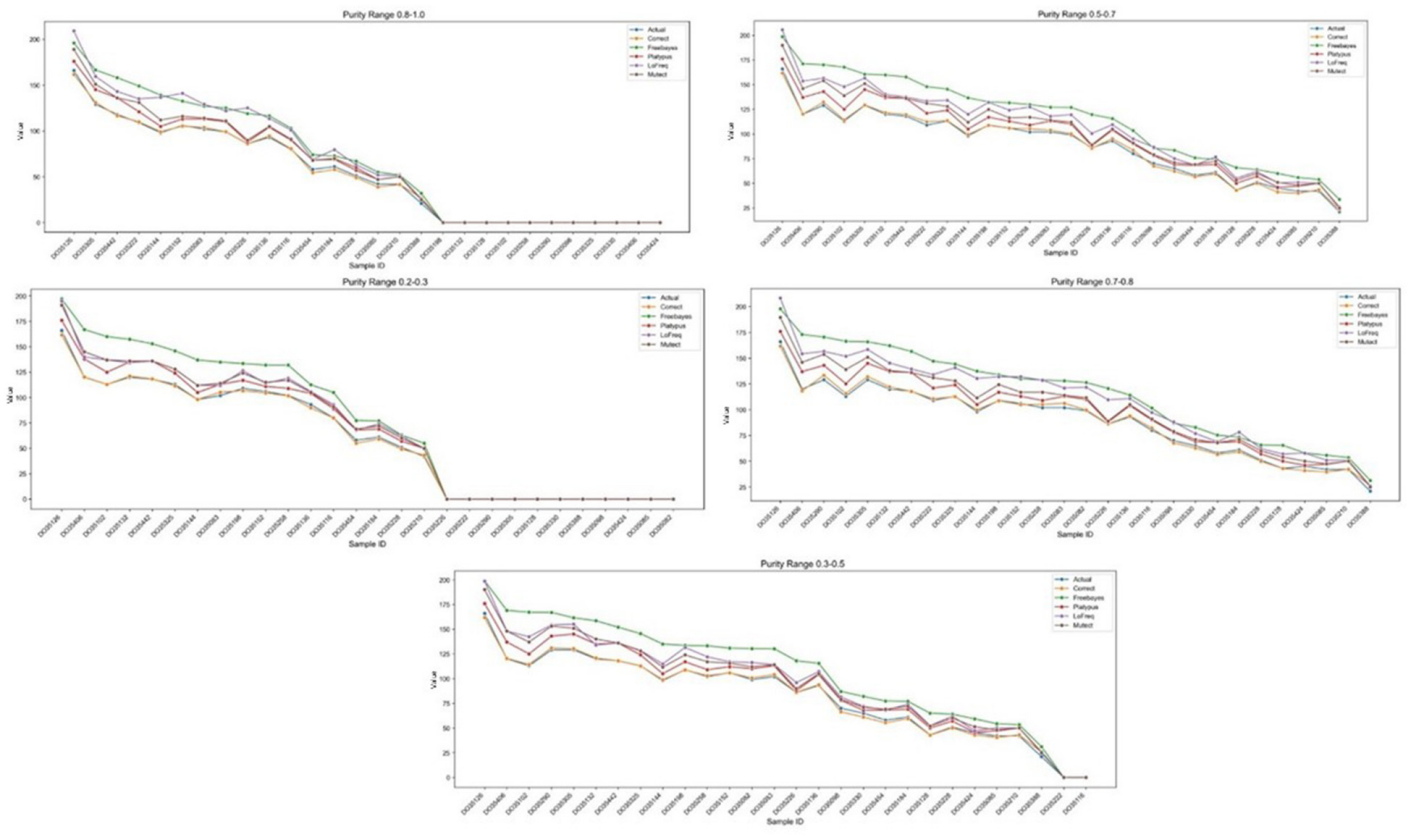
Performance benchmarking against mainstream variant callers across varying sample purities. The subplots display the SNV detection accuracy of our proposed framework (Correct) compared to standard callers including Freebayes, Platypus, LoFreq, and Mutect. The evaluation spans five distinct host purity ranges: 0.8–1.0, 0.7–0.8, 0.5–0.7, 0.3–0.5, and the ultra-low purity range of 0.2–0.3. Our corrected results consistently demonstrate the highest concordance with the Actual baseline, exhibiting exceptional robustness particularly in highly heterogeneous (low-purity) settings.

The comparative results are presented in Figure 3. In high-purity scenarios (e.g., 0.8–1.0), most callers performed reasonably well, though our model still maintained the closest proximity to the actual count baseline. However, as the host purity decreased, the performance of traditional callers deteriorated rapidly. Notably, in the ultra-low purity range (0.2–0.3), mainstream tools like Freebayes and Platypus exhibited severe deviations, struggling to distinguish true host variants from microbial background noise and sequencing artifacts. In stark contrast, our Transformer-based correction model exhibited exceptional robustness, consistently tracking the “Actual” baseline across all purity spectrums. This demonstrates the model's superior capability in filtering out noise in highly complex, low-purity symbiotic samples.

### 3.3 Residual distribution and model unbiasedness

To further assess the statistical reliability of our correction framework, we analyzed the distribution of the correction residuals, defined as the difference between the actual SNV counts and the model's corrected SNV counts (Residual = Actual SNV – Corrected SNV).

**Figure 4:**
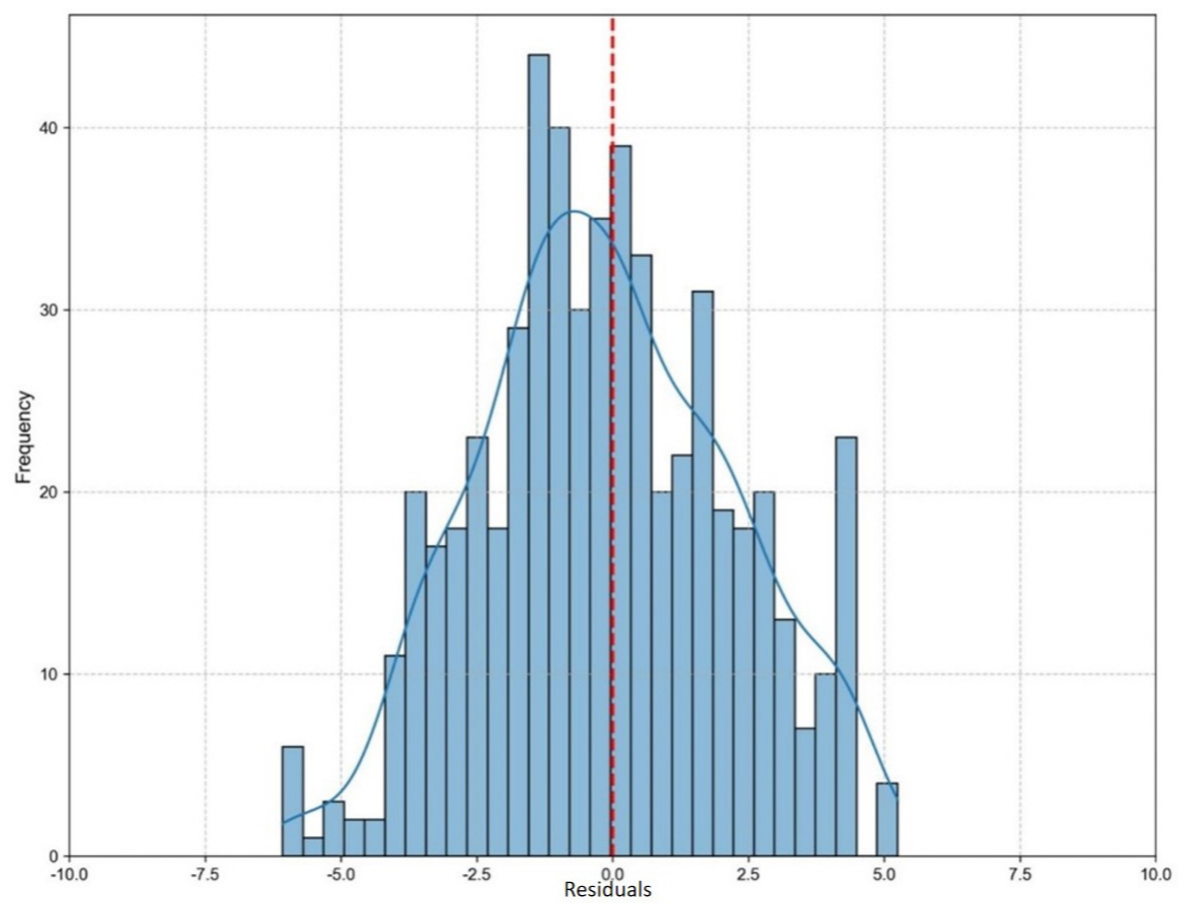
Distribution of correction residuals. The histogram displays the frequency of correction errors, calculated as the difference between the actual SNV counts and the model's corrected SNV counts. The normal-like distribution tightly centered around zero (indicated by the red dashed line) confirms the unbiased nature of the framework's predictions, ensuring no systematic overestimation or underestimation.

As shown in Figure 4, the histogram of the residuals closely follows a normal distribution centered around zero (indicated by the red dashed line). The absence of significant skewness to either the left or the right confirms that our model does not introduce new systematic biases (i.e., it does not systematically overcorrect or undercorrect). This unbiased, zero-centered error distribution is critical for downstream biological analyses, ensuring that the corrected genomic profiles provide a statistically sound foundation for studying host-microbe genetic interactions.

### 3.4 Robustness of correction along purity variations

To visualize how the SNV detection stability correlates with sample purity, we plotted the relationship between the host sample purity intervals and the mean SNV counts. In this analysis, the mean SNV count within a specific purity interval was used as the *y*-axis coordinate for each data point.

**Figure 5:**
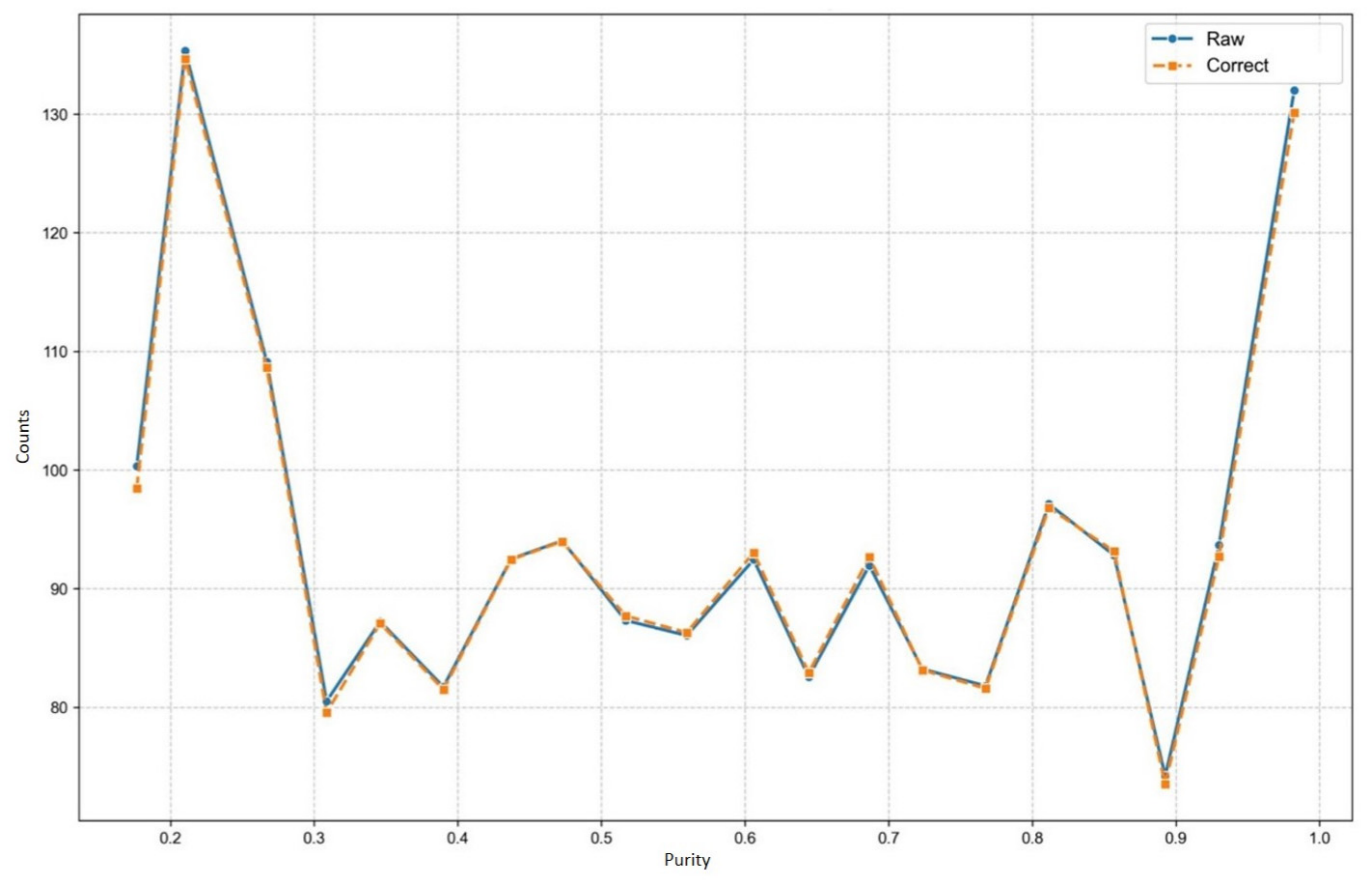
Stability of SNV correction across the host purity spectrum. The plot visualizes the dynamic relationship between varying host sample purity levels (*x*-axis, ranging from 0.2 to 1.0) and the mean SNV counts (*y*-axis). The corrected trajectory (orange dashed line) tightly tracks the variance of the raw input dynamics (blue solid line), demonstrating the model's adaptive capacity to preserve true biological signals while filtering out artifacts across the entire purity gradient.

Figure 5 displays the dynamic trend curves. The corrected values (Correct) maintain a highly consistent and tight trajectory alongside the raw input data dynamics across the entire purity spectrum (from 0.2 to 1.0). This demonstrates that the model is not merely applying a static mathematical suppression but is dynamically adapting to the intrinsic data variance at different purity levels. The framework successfully preserves the true biological signal variations while neutralizing the purity-induced artifacts.

### 3.5 Feature correlation and rationale of the multi-dimensional input

The strong performance of our framework relies heavily on the comprehensive Feature Extraction Module. To validate the informational value and independence of our selected features, we conducted a correlation analysis within the five distinct feature groups: Repeat Region, DNase Region, Purity Basic, Depth & VAF Stats, and Blacklist Region.

**Figure 6:**
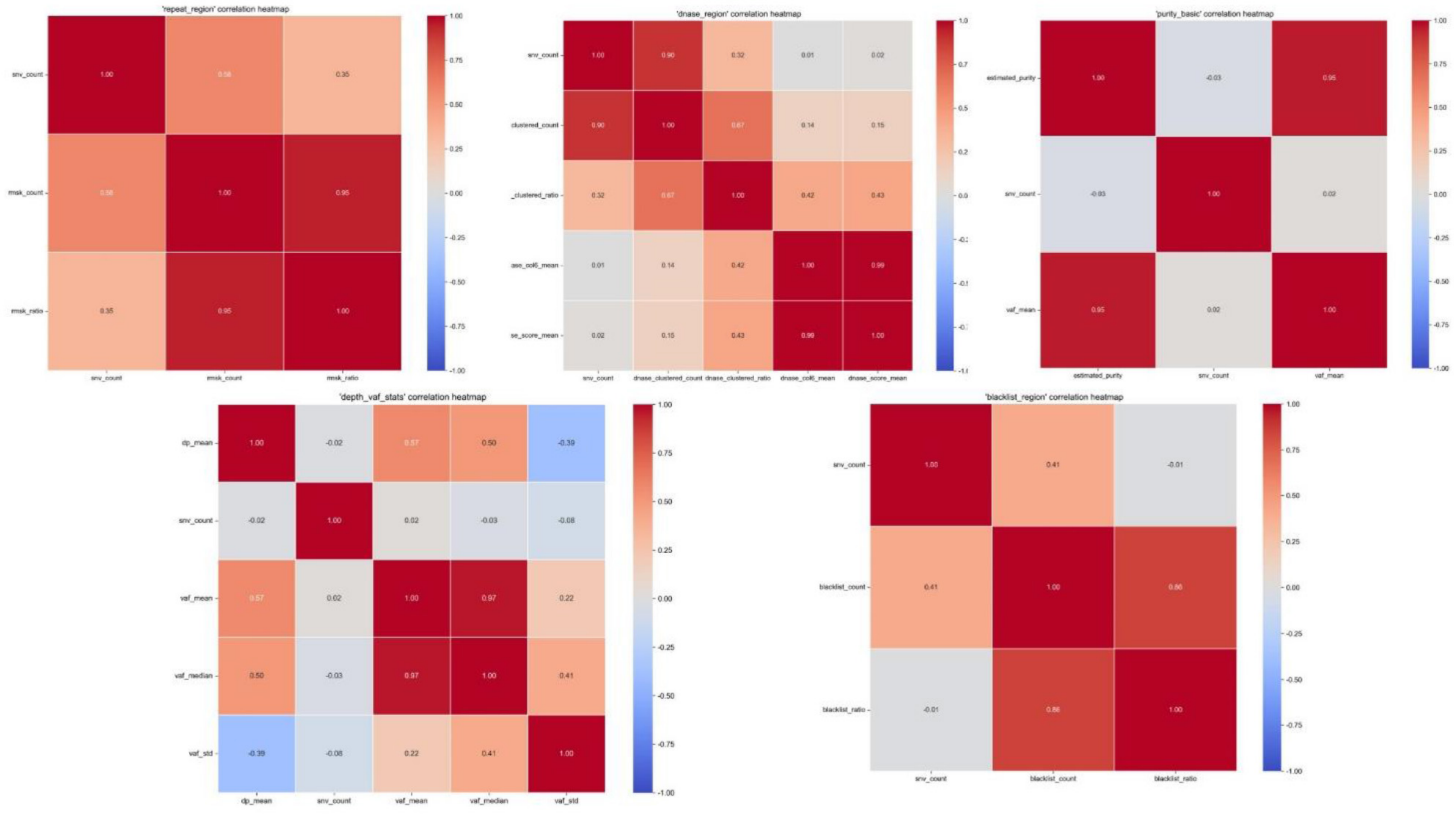
Correlation analysis of the extracted multi-dimensional feature groups. The heatmaps represent the intra-group Pearson correlations for the five distinct feature sets utilized by the model: Repeat Region, DNase Region, Purity Basic, Depth & VAF Stats, and Blacklist Region. The clear and biologically interpretable correlation patterns validate the independence and rationale of the feature selection and grouping strategy, which prevents confounding and enhances the Transformer's encoding efficiency.

The correlation heatmaps are presented in Figure 6. The analysis reveals clear intra-group correlations that align with biological and technical expectations (e.g., strong correlations between VAF mean and VAF median within the “depth_vaf_stats” group). Furthermore, the distinct heatmap patterns across the five groups validate our feature selection and grouping strategy. By compartmentalizing these diverse signals—ranging from mappability issues (blacklist/repeat regions) to chromatin accessibility (DNase) and basic sequencing metrics (depth/purity)—the model avoids feature confounding. This structured multi-dimensional input allows the Group Encoder and Transformer Decoder to effectively weigh and integrate distinct profiles, ultimately empowering the precise correction of SNV counts in complex mixed ecosystems.

## 4 Discussion

The study of host-microbe symbioses has increasingly relied on high-throughput sequencing to unravel the complex genetic and metabolic interactions that underpin ecosystem robustness. However, computational tools have historically lagged behind sequencing technologies, particularly when dealing with the extreme heterogeneity of mixed-species samples. In this study, we introduced a novel Transformer-based framework specifically designed to correct single nucleotide variant (SNV) counts in low-purity host environments, a common yet highly challenging scenario in microbiome research.

Our results demonstrate a significant leap in performance over traditional variant callers. Conventional tools like Freebayes, Platypus, and Mutect rely heavily on fixed statistical priors and probabilistic models optimized for relatively pure or clonal populations. As evidenced by our benchmarking results (Figure 3), these tools suffer catastrophic performance drops in ultra-low purity settings (e.g., 0.2–0.3). In these highly heterogeneous environments, authentic host minor alleles are easily swamped by microbial genomic noise or sequencing artifacts, leading to severe misquantification. Our deep learning framework overcomes this by utilizing a multi-dimensional feature extraction strategy. By evaluating VAF statistics alongside genomic context elements like repeat regions, DNase hypersensitivity, and depth profiles, the model does not merely look at base quality; it contextualizes the variant within the structural and functional landscape of the genome. The Group Encoder and Transformer Decoder effectively learn the complex, non-linear error distributions that traditional statistical models fail to capture.

The biological implications of this computational advancement for symbiosis research are substantial. Accurately quantifying host SNVs is the fundamental first step in conducting precise Genome-Wide Association Studies (GWAS) or expression quantitative trait loci (eQTL) analyses within symbiotic microenvironments (such as the gut mucosa or plant-root interfaces). By neutralizing purity-induced biases and ensuring an unbiased residual distribution (Figure 4), our tool ensures that the host's genetic background can be reliably assessed even when microbial loads are overwhelmingly high. This empowers researchers to confidently link specific host genetic variations to microbial community assembly, immune tolerance, and metabolic cross-talk, thereby answering critical questions about how partner selection and host genetics set resilience thresholds after ecological disturbances.

Despite the promising results, this study has limitations that warrant future exploration. Currently, the framework's efficacy has been extensively validated using mathematically simulated datasets that mimic heterogeneous biological mixtures. While these simulations rigorously control for ground truth, real-world biological samples may introduce unforeseen artifacts, such as severe DNA degradation or extreme host-microbe sequence homologies. Future iterations of this tool will focus on validating and fine-tuning the model using deeply sequenced biological cohorts with matched single-cell or pure-tissue controls. Additionally, expanding the framework to simultaneously correct microbial structural variants (SVs) or insertions/deletions (InDels) would further enrich the analytical toolkit available to microbial ecologists.

## 5 Conclusion

In conclusion, we have developed and validated a state-of-the-art, Transformer-based computational framework for the accurate correction of single nucleotide variant (SNV) detection in heterogeneous host-microbiome samples. By innovatively integrating multi-dimensional genomic features and leveraging deep learning architectures, the model successfully overcomes the limitations of traditional variant callers, demonstrating exceptional robustness and unbiased accuracy across a wide spectrum of sample purities, particularly in ultra-low purity conditions. This tool directly addresses a critical methodological bottleneck in symbiosis research, providing a reliable genomic profiling solution that will facilitate deeper investigations into host-microbe interactions, genetic feedback loops, and the molecular foundations of ecosystem stability.

## Data Availability

The original contributions presented in the study are included in the article/supplementary material, further inquiries can be directed to the corresponding authors.
